# FungalBraid 2.0: expanding the synthetic biology toolbox for the biotechnological exploitation of filamentous fungi

**DOI:** 10.3389/fbioe.2023.1222812

**Published:** 2023-08-07

**Authors:** Elena Moreno-Giménez, Mónica Gandía, Zara Sáez, Paloma Manzanares, Lynne Yenush, Diego Orzáez, Jose F. Marcos, Sandra Garrigues

**Affiliations:** ^1^ Food Biotechnology Department, Instituto de Agroquímica y Tecnología de Alimentos (IATA), Consejo Superior de Investigaciones Científicas (CSIC), Valencia, Spain; ^2^ Instituto de Biología Molecular y Celular de Plantas (IBMCP), Consejo Superior de Investigaciones Científicas (CSIC)-Universitat Politècnica de València (UPV), Valencia, Spain; ^3^ Preventive Medicine and Public Health, Food Science, Toxicology and Forensic Medicine Department. Faculty of Pharmacy. Universitat de València. Vicente Andrés Estellés s/n, Valencia, Spain

**Keywords:** fungal synthetic biology, GoldenBraid, promoters, selection markers, luciferase-based reporter system, CRISPR activation, filamentous fungi

## Abstract

Fungal synthetic biology is a rapidly expanding field that aims to optimize the biotechnological exploitation of fungi through the generation of standard, ready-to-use genetic elements, and universal syntax and rules for contributory use by the fungal research community. Recently, an increasing number of synthetic biology toolkits have been developed and applied to filamentous fungi, which highlights the relevance of these organisms in the biotechnology field. The FungalBraid (FB) modular cloning platform enables interchangeability of DNA parts with the GoldenBraid (GB) platform, which is designed for plants, and other systems that are compatible with the standard Golden Gate cloning and syntax, and uses binary pCAMBIA-derived vectors to allow *Agrobacterium tumefaciens*-mediated transformation of a wide range of fungal species. In this study, we have expanded the original FB catalog by adding 27 new DNA parts that were functionally validated *in vivo*. Among these are the resistance selection markers for the antibiotics phleomycin and terbinafine, as well as the uridine-auxotrophic marker *pyr4.* We also used a normalized luciferase reporter system to validate several promoters, such as P*pkiA*, P*7760*, P*ef1*α, and P*afpB* constitutive promoters, and P*glaA*, P*amyB*, and P*xlnA* inducible promoters. Additionally, the recently developed dCas9-regulated GB_SynP synthetic promoter collection for orthogonal CRISPR activation (CRISPRa) in plants has been adapted in fungi through the FB system. In general, the expansion of the FB catalog is of great interest to the scientific community since it increases the number of possible modular and interchangeable DNA assemblies, exponentially increasing the possibilities of studying, developing, and exploiting filamentous fungi.

## 1 Introduction

Filamentous fungi have acquired a great biotechnological relevance as biofactories for the sustainable production of organic acids, proteins, enzymes, and metabolites with applications in the agri-food, chemical, pharmaceutical, textile, paper, and biofuel industries (Meyer et al., 2016). Their ability to grow on many distinct and economic substrates and plant residues, and their high secretory capacity justify the biotechnological interest of these microorganisms, which have become essential contributors to the so-called circular bio-economy (Meyer et al., 2020). Enzymes produced by fungi currently make up more than half of the enzymes used in the industry (de Vries et al., 2020). Additionally, fungal genomes contain a large number of biosynthetic gene clusters encoding potentially useful biomolecules to be exploited (Robey et al., 2021), reflecting the relevance of filamentous fungi as cell factories. However, there are still aspects that need to be improved since the conditions and levels of production of different biomolecules are highly variable, and some of them are difficult to produce in a cost-efficient manner.

Synthetic biology (SynBio) is an ever-expanding scientific field that has revolutionized genetic and metabolic engineering. SynBio provides new tools for the generation of ready-to-use, standardized, modular genetic elements to obtain microbial strains with optimized properties either by the production of specific proteins or by fine-tuning the expression of specific metabolic pathway-related genes (Benner and Sismour, 2005). In this context, fungal SynBio is rapidly evolving. Our group has adapted the GoldenBraid (GB) modular cloning platform originally developed for plants (Sarrion-Perdigones et al., 2013) to filamentous fungi, a variant called FungalBraid (FB) (https://gbcloning.upv.es/fungal/). This modular cloning method is based on type IIS restriction enzymes and pCAMBIA-derived binary vectors for *Agrobacterium tumefaciens*-mediated transformation (ATMT), with the main advantages of the full reusability of its DNA parts and their interchangeability between plants and fungi as long as they are functionally compatible (Hernanz-Koers et al., 2018; Vazquez-Vilar et al., 2020). The domestication or incorporation of new DNA parts into the FB system is achieved by cloning them into level 0 pUPD2 vectors, and transcriptional units (TUs) are then formed by combining different level 0 parts in a multipartite assembly into level 1 pDGB3α vectors. The GB and FB systems allow the combination of different TUs contained in two compatible pDGB3α vectors in a bipartite assembly into level 2 pDGB3Ω vectors, which can then be combined in the same way back into pDGB3α vectors, allowing the indefinite expansion of the multigene construct and designs of increased complexity.

Since the development of the FB system, an increasing number of SynBio-based applications in fungi have been reported (Dahlmann et al., 2021; Mózsik et al., 2021; Mózsik et al., 2022), which highlights the need for a boost in the SynBio toolkit for these organisms. However, there is still a shortage of tools for orthogonal and fine-tuned expression of genes applied to filamentous fungi. In this sense, an increase in the repertoire of promoters is required. Promoters with different expression levels or which are inducible and/or cell-specific would increase the flexibility and the ability to optimize expression systems, especially for proteins which can be toxic. These promoters may come from different organisms or may be created using synthetic designs. Considering that constitutive and inducible promoters are commonly used among the scientific community, synthetic promoters have been less exploited. These promoters often comprised a core or minimal promoter and an upstream region in which cis-regulatory elements are incorporated (Martins-Santana et al., 2018). These cis-regulatory elements are typically obtained from the binding sites of transcriptional regulators which activate or inactivate gene expression. Although natural transcriptional regulators limit the freedom in the design of cis-regulatory elements, the use of CRISPR activation (CRISPRa) strategies allows the use of virtually any 20-base pair (bp) sequence as a cis-regulatory box in the development of synthetic promoters (Moreno-Giménez et al., 2022). In this regard, the collection of nuclease-deactivated Cas9 (dCas9)-regulated synthetic promoter GB_SynP that has been recently developed for plants (Moreno-Giménez et al., 2022) could easily be adapted to fungi, given the interchangeability of DNA parts between GB and FB systems. Additionally, GB/FB systems provide a standard measurement using a luciferase/renilla transient assay to estimate relative expression levels of promoters, including the synthetic promoters (Vazquez-Vilar et al., 2017; Gandía et al., 2022).

In this study, we have incorporated 27 new genetic parts into the FB system, which include native strong and inducible fungal promoters, synthetic promoters, terminators, and selection markers. All these components have been validated *in vivo* in two economically relevant fungi: the non-model postharvest pathogen of citrus *Penicillium digitatum* (Palou, 2014), and in the well-known fungus with the Generally Recognized as Safe (GRAS) status and a long record of industrial use *Penicillium chrysogenum* (Fierro et al., 2022)*.* The strength of the constitutive promoters has been characterized and compared in a nanoluciferase-normalized luciferase-based reporter system; the induction levels of the inducible promoters have also been quantified, and the activation of the synthetic promoters has been studied using programmable transcriptional factors based on CRISPRa (Mózsik et al., 2021). Overall, the expansion of the FB toolkit will be of great interest to the scientific community to further aid the exploitation of fungal workhorses and accelerate the discovery and production of (novel) bioactive molecules for multiple biotechnological applications.

## 2 Materials and methods

### 2.1 Strains, media, and growth conditions

The fungal strains used in this study were *P. digitatum* CECT 20796 (isolate PHI26) (Marcet-Houben et al., 2012) and *P. chrysogenum* wild-type ATCC 10002 (Q176) (Hegedüs et al., 2011). Fungi were routinely cultured on potato dextrose agar (PDA, Difco-BD Diagnostics) plates for 7 days at 25°C. For transformation, the generated vectors were amplified in the bacterium *Escherichia coli* JM109 and grown in Luria–Bertani (LB) medium at 37°C with either 50 μg/mL chloramphenicol, 50 μg/mL kanamycin, 100 μg/mL spectinomycin, or 100 μg/mL ampicillin depending on the vector. *A. tumefaciens* AGL-1 was cultured in LB medium at 28°C with 20 μg/mL rifampicin and the corresponding antibiotic depending on the vector used.

For growth profiles, 5 µL of conidial suspension (5 × 10^4^ conidia/mL) was deposited on the center of PDA plates, and colony morphology was assessed and compared daily by visual inspection.

### 2.2 Design, domestication, and DNA assemblies of genetic elements

All the genetic elements that have been incorporated on the FB platform are listed in Table 1. New DNA parts were domesticated according to GB rules and tools (https://gbcloning.upv.es) and ordered from an external company as synthetic genes (gBlocks™, IDT). In the case of the coding sequence (CDS) of *pyr*4, the gene from *Trichoderma reesei* was codon-optimized according to the optimal codon frequency of *Penicillium* genera prior to GB/FB domestication. Domesticated elements were ligated into the pUPD2 entry vector via the restriction–ligation protocol, as previously described (Hernanz-Koers et al., 2018; Vazquez-Vilar et al., 2020). Positive *E. coli* clones were confirmed by routine PCR amplifications and Sanger sequencing using external specific primers OJM524 and OJM525, which are designed for pUPD2 vectors (Hernanz-Koers et al., 2018) (Table 2). Multiple assemblies into pDGB3α vectors of the DNA parts contained in pUPD2 vectors were carried out to obtain different TUs, and binary assemblies were subsequently performed to combine different TUs into multigenetic constructs within these pDGB3α or pDGB3Ω vectors, as previously described (Hernanz-Koers et al., 2018; Vazquez-Vilar et al., 2020).

**TABLE 1:** FB parts reported in this study. DNA parts are grouped according to the purpose for which they were used.

Selection marker				
Auxotrophy				
**Code**	**Name**	**Plasmid**	**Description**	**Reference**
FB271*	P*pyr4*	pUPD2	Promoter of the *pyr4* gene from *T. reesei*	This study
FB272*	*pyr4*	pUPD2	Coding sequence of the *pyr4* gene from *T. reesei*	This study
FB273*	T*pyr4*	pUPD2	Terminator of the *pyr4* gene from *T. reesei*	This study
FB293*	TU_*pyr4*	pDGB3α2	Assembly of the transcriptional unit for the auxotrophy marker *pyr4* from *T. reseei*	This study
FB359*	5′ upstream Pdig *pyrG*	pUPD2	5′ upstream region of the *pyrG* gene in *P. digitatum*	This study
FB361*	3′ downstream Pdig *pyrG*	pUPD2	3′ downstream region of the *pyrG* gene in *P. digitatum*	This study
FB372*	FB359+FB361	pDGB3α1	Assembly for *pyrG* deletion in *P. digitatum*	This study
Resistance				
**Code**	**Name**	**Plasmid**	**Description**	**Reference**
FB413*	*ble*	pUPD2	Coding sequence for phleomycin resistance	This study
FB414*	*ergA*	pUPD2	Coding sequence for terbinafine resistance	This study
FB411*	P*pcbC*	pUPD2	Promoter of isopenicillin N synthase from *P. rubens*	This study
FB416*	T*amdS*	pUPD2	Terminator of acetamidase-encoding gene *amdS* from *A. nidulans*	This study
FB430*	P*pcbc:ble:*T*amdS*	pDGB3α2	TU for the expression of phleomycin resistance	This study
FB431*	P*gpdA:ergA:*T*amdS*	pDGB3α2	TU for the expression of terbinafine resistance	This study

**Constitutive/inducible promoters**				
**Code**	**Name**	**Plasmid**	**Description**	**Reference**
FB007	P*gpdA*	pUPD2	Promoter of glyceraldehyde-3-phosphate dehydrogenase from *A. nidulans*	Hernanz-Koers et al. (2018)
FB291*	P*xlnA*	pUPD2	Promoter of the endo-1,4-beta-xylanase A gene from *A. nidulans*, xylose-inducible	This study
FB389*	P*pkiA*	pUPD2	Promoter of the highly expressed pyruvate kinase gene from *A. niger*	This study
FB404*	P*afpB*	pUPD2	Promoter of the antifungal protein *afpB* gene from *P*. *digitatum*	This study
FB405*	P*glaA*	pUPD2	Promoter of the glucoamylase gene from *A. niger,* maltose/starch-inducible	This study
FB406*	P*amyB*	pUPD2	Promoter of the TAKA-amylase A gene from *A. oryzae,* maltose/starch-inducible	This study
FB407*	P*ef1α*	pUPD2	Promoter of the elongation factor 1-α gene from *P. digitatum* (PDIG_59570)	This study
FB408*	P07760	pUPD2	Promoter of the ubiquitin ligase gene from *P. digitatum* (PDIG_07760)	This study
GB0096	Luciferase (Luc)	pUPD	Coding sequence for the firefly luciferase protein	Sarrion-Perdigones et al. (2013)
FB001	P*trpC*	pUPD2	Promoter of the multifunctional tryptophan biosynthesis protein coding gene *trpC* from *A. nidulans*	Hernanz-Koers et al. (2018)
FB002	T*tub*	pUPD2	Terminator of the tubulin-encoding gene from *N. crassa*	Hernanz-Koers et al. (2018)
FB008	T*trpC*	pUPD2	Terminator of the *trpC* gene from *A. nidulans*	Hernanz-Koers et al. (2018)
FB009	P*trpC*:*nptII*:T*tub*	pDGB3α2	TU for the expression of geneticin resistance	Hernanz-Koers et al. (2018)
FB312	P*gpdA*:Nluc:T*tub*	pDGB3α1R	TU for the expression of nanoluciferase (Nluc) under P*gpdA*	Gandía et al. (2022)
FB323	P*gpdA*:Nluc:T*tub::*P*trpC:nptII:*T*tub:: Ppaf:*Luc*:Ttub*	pDGB3α1	Module for the expression of geneticin resistance and nanoluciferase, and the expression of firefly luciferase under the P*paf*	Gandía et al. (2022)
FB367*	P*gpdA*:Nluc:T*tub::*P*trpC:nptII:*T*tub*	pDGB3α1	Module for the expression of geneticin resistance and nanoluciferase	This study
FB417	P*afpB*:Luc:T*trpC*	pDGB3α2	TU for the expression of luciferase under P*afpB*	This study
FB418	P*glaA*:Luc:T*trpC*	pDGB3α2	TU for the expression of luciferase under P*glaA*	This study
FB419	P*amyB*:Luc:T*trpC*	pDGB3α2	TU for the expression of luciferase under P*amyB*	This study
FB420	P*ef1α*:Luc:T*trpC*	pDGB3α2	TU for the expression of luciferase under P*ef1α*	This study
FB421	P07760:Luc:T*trpC*	pDGB3α2	TU for the expression of luciferase under P07760	This study
FB423	P*gpdA*:Luc:T*trpC*	pDGB3α2	TU for the expression of luciferase under P*gpdA*	This study
FB424	P*xlnA*:Luc:T*trpC*	pDGB3α2	TU for the expression of luciferase under P*xlnA*	This study
FB426	P*pkiA*:Luc:T*trpC*	pDGB3α2	TU for the expression of luciferase under P*pkiA*	This study
FB432	FB367+FB417	pDGB3Ω1	Module for the expression of geneticin resistance, Nluc under the P*gpdA* promoter, and luciferase under P*afpB*	This study
FB433	FB367+FB418	pDGB3Ω1	Module for the expression of geneticin resistance, Nluc under the P*gpdA* promoter, and luciferase under P*glaA*	This study
FB434	FB367+FB419	pDGB3Ω1	Module for the expression of geneticin resistance, Nluc under the P*gpdA* promoter, and luciferase under P*amyB*	This study
FB435	FB367+FB420	pDGB3Ω1	Module for the expression of geneticin resistance, Nluc under P*gpdA* promoter, and luciferase under P*ef1α*	This study
FB436	FB367+FB421	pDGB3Ω1	Module for the expression of geneticin resistance, Nluc under the P*gpdA* promoter, and luciferase under P07760	This study
FB438	FB367+FB423	pDGB3Ω1	Module for the expression of geneticin resistance, Nluc under the P*gpdA* promoter, and luciferase under P*gpdA*	This study
FB439	FB367+FB424	pDGB3Ω1	Module for the expression of geneticin resistance, Nluc under the P*gpdA* promoter, and luciferase under P*xlnA*	This study
FB441	FB367+FB426	pDGB3Ω1	Module for the expression of geneticin resistance, Nluc under the P*gpdA* promoter, and luciferase under P*pkiA*	This study

**dCas9-activated synthetic promoters**				
**Code**	**Name**	**Plasmid**	**Description**	**Reference**
GB2815	RandomSequence R1	pUPD2	Random sequence R1 of 1240 bp for the A1 distal promoter position	Moreno-Giménez et al. (2022)
GB2878	G1aG2b.1	pUPD2	A2 proximal promoter sequence containing the target sequence for gRNA1 flanked by random sequences	Moreno-Giménez et al. (2022)
GB2885	G1ab.1	pUPD2	A2 proximal promoter sequence consisting of two times the target sequence for gRNA1 flanked by random sequences	Moreno-Giménez et al. (2022)
GB3276	G1abc.3	pUPD2	A2 proximal promoter sequence containing three times the target sequence for gRNA1 flanked by random sequences	Moreno-Giménez et al. (2022)
GB3413	m*PAF*	pUPD2	Minimal promoter of the *paf* gene from *P. chrysogenum*, containing 62 bp upstream the transcription start site and the 5′UTR region	Moreno-Giménez et al. (2022)
FB395*	R1:G1aG2b.1:m*PAF*:Luc:T*trpc*	pDGB3α2	TU for the expression of luciferase under a synthetic promoter containing the target sequence for gRNA1 (1xLuc)	This study
FB396*	R1:G1ab.1:m*PAF*:Luc:T*trpc*	pDGB3α2	TU for the expression of luciferase under a synthetic promoter containing two times the target sequence for gRNA1 (2xLuc)	This study
FB397*	R1:G1abc.3:m*PAF*:Luc:T*trpc*	pDGB3α2	TU for the expression of luciferase under a synthetic promoter containing three times the target sequence for gRNA1 (3xLuc)	This study
FB398*	FB367+FB395	pDGB3Ω1	Module for the expression of geneticin resistance, Nluc under the P*gpdA* promoter, and luciferase under a synthetic promoter containing the target sequence for gRNA1	This study
FB399*	FB367+FB396	pDGB3Ω1	Module for the expression of geneticin resistance, Nluc under the P*gpdA* promoter, and luciferase under a synthetic promoter containing two times the target sequence for gRNA1 (2xLuc)	This study
FB400*	FB367+FB397	pDGB3Ω1	Module for the expression of geneticin resistance, Nluc under the P*gpdA* promoter, and luciferase under a synthetic promoter containing three times the target sequence for gRNA1 (3xLuc)	This study
FB403	pAMA18.0_gRNA1	pAMA18.0	Expression plasmid for the dCas9 activation system and the (GB_SynP) gRNA1	This study

*DNA parts from this study which are deposited in Addgene.

**TABLE 2:** Primers used in this study.

ID	Use*	Sequence 5′-3′**	Tm (°C)	Origin	Purpose	Reference
OJM371	F	ATA​GAT​CTA​ACT​GAT​ATT​GAA​GGA​GCA	52	P*trpC*	Molecular characterization	This study
OJM509	F	GCGCCGTCTCGCTCGGGAGTGGCGCATGCGGACAGACGG	64	P*gpdA*	Molecular characterization	Hernanz-Koers et al. (2018)
OJM522	R	GCG​CCG​TCT​CGC​TCA​AGC​GCA​TGT​CTC​AGA​CGGTCGATG	62	T*trpC*	Molecular characterization	Hernanz-Koers et al. (2018)
OJM524	F	GCT​TTC​GCT​AAG​GAT​GAT​TTC​TGG	70	pUPD2	Molecular characterization	Hernanz-Koers et al. (2018)
OJM525	R	CAG​GGT​GGT​GAC​ACC​TTG​CC	66	pUPD2	Molecular characterization	Hernanz-Koers et al. (2018)
OJM555	R	TCA​TCA​TGC​AAC​ATG​CAT​GTA	58	T*tub*	Molecular characterization	Hernanz-Koers et al. (2018)
OJM655	R	CAT​CCA​TAC​TCC​ATC​CTT​CCC	60	pAMA18.0	Molecular characterization and sequencing	This study
OJM656	F	CAT​TTT​TGT​CGT​CAT​GTG​CTG​G	55	5′ Pdig *pyrG*	Molecular characterization	This study
OJM657	R	GAA​GGC​TGA​ACT​CAC​TGT​GG	55	3′ Pdig *pyrG*	Molecular characterization	This study
OJM662	F	GCT​TTT​GCT​AAC​CAT​TTG​GGA​CAC	52	GB B6 code	Cloning of FB372 into pDGB3α1	This study
OJM663	R	AGC​GGT​GTC​CCA​AAT​GGT​TAG​CAA	52	GB C1 code	Cloning of FB372 into pDGB3α1	This study
OJM696	F	TTG​TCT​CAC​TCT​CTC​TTT​TCC	51	*pyr4*	Molecular characterization	This study
OJM697	R	ATT​CCA​TGC​TTC​CAG​ATC​C	51	*pyr4*	Molecular characterization	This study
OJM698	F	ATGGTCTCACCGACCAGTCCTGATGAGTCCGTGAGGACGAAACGAG	60	pAMA18.0	Cloning of gRNA1 into pAMA18.0	This study
OJM699	R	ATGGTCTCTAAACTCTTCTCTCACCAACCAGTC GACGAGCTTACTCGTTTCGTCCTCACGGACTCA	60	pAMA18.0	Cloning of gRNA1 into pAMA18.0	This study
OJM705	F	TCC​TGG​AAG​TGC​GTT​GAT​CA	51	P*xlnA*	Molecular characterization	This study
OJM706	F	GGA​AGA​GAA​AAC​CTC​CGA​GTA​C	54	P*pkiA*	Molecular characterization	This study
OJM707	F	ATG​AAT​TCC​ACC​GAA​TGC​AC	53	P*afpB*	Molecular characterization	This study
OJM708	F	TGC​CAT​TGG​CGG​AGG​GGT​CC	53	P*glaA*	Molecular characterization	This study
OJM709	F	TCA​ACT​GAT​TAA​AGG​TGC​CG	53	P*amyB*	Molecular characterization	This study
OJM710	F	GTG​AAA​AAA​CGG​ATG​GGG​AC	53	P*ef1α*	Molecular characterization	This study
OJM711	F	GAT​AAT​GGT​GAT​TCG​GCG​CG	53	P07760	Molecular characterization	This study
OJM715	F	GTA​TCT​GCA​TGT​TGC​ATC​GG	53	P*pcbC*	Molecular characterization	This study
OJM716	R	TAC​CGC​TCG​TAC​CAT​GGG​TT	53	T*amds*	Molecular characterization	This study

*F, forward; R, reverse.

**gRNA1 sequence is underlined.

The sequence of the single-guide RNA 1 (gRNA1) required to activate GB_SynP promoters was checked for the absence of off-target mutations in the *P. digitatum* genome using Geneious Prime software (https://www.geneious.com/), and was cloned into the pAMA18.0 vector, as described previously (Mózsik et al., 2021). Briefly, a primer pair was designed (OJM698 and OJM699, Table 2) which contained the target sequence of gRNA1, the hammerhead ribozyme, the inverted repetition of the 5′-end of the spacer sequence, and the recognition sites of *Bsa*I. The resulting PCR product was purified (Wizard SV Gel and PCR Clean-Up System, Promega) and inserted into pAMA18.0 via the restriction–ligation reaction with *Bsa*I and T4 ligase. Correct assemblies of the resulting pAMA18.0_gRNA1 vector were confirmed by Sanger sequencing.

### 2.3 Fungal transformation and mutant confirmation

Transformation of *P. digitatum* CECT 20796 (PHI26) and *P. chrysogenum* ATTC 10002 (Q176) with the corresponding FB binary vectors described in Table 1 was performed through ATMT, as previously described (Khang et al., 2007), with some modifications (Harries et al., 2015; Vazquez-Vilar et al., 2020).

In the case of *P. digitatum* uridine-auxotrophic Δ*pyrG* mutants, in which the *pyrG* gene (gene ID PDIG_38390) was deleted by homologous recombination without the insertion of any positive selection marker, mutants were selected on PDA supplemented with 1.22 g/L uridine (Sigma-Aldrich) and 1.25 g/L of 5-fluoroorotic acid (5-FOA, Formedium). For the ectopically complemented *P. digitatum* Δ*pyrG:pyr4* strains, mutants were selected on PDA plates. *P. digitatum* and *P. chrysogenum* ectopic transformants containing *ble* TU (phleomycin^R^) were selected on PDA plates supplemented with 35 μg/mL and 25 μg/mL phleomycin (InvivoGen), respectively. Finally, *P. chrysogenum* ectopic transformants carrying *ergA* TU (terbinafine^R^) were selected on PDA plates supplemented with 0.5 μg/mL terbinafine hydrochloride (Sigma-Aldrich).

*P. digitatum* and *P. chrysogenum* transformants carrying different luciferase reporter constructs to test the constitutive, inducible, and GB_SynP promoters were selected on PDA plates containing 25 μg/mL geneticin (G418) (InvivoGen).

**FIGURE 1:**
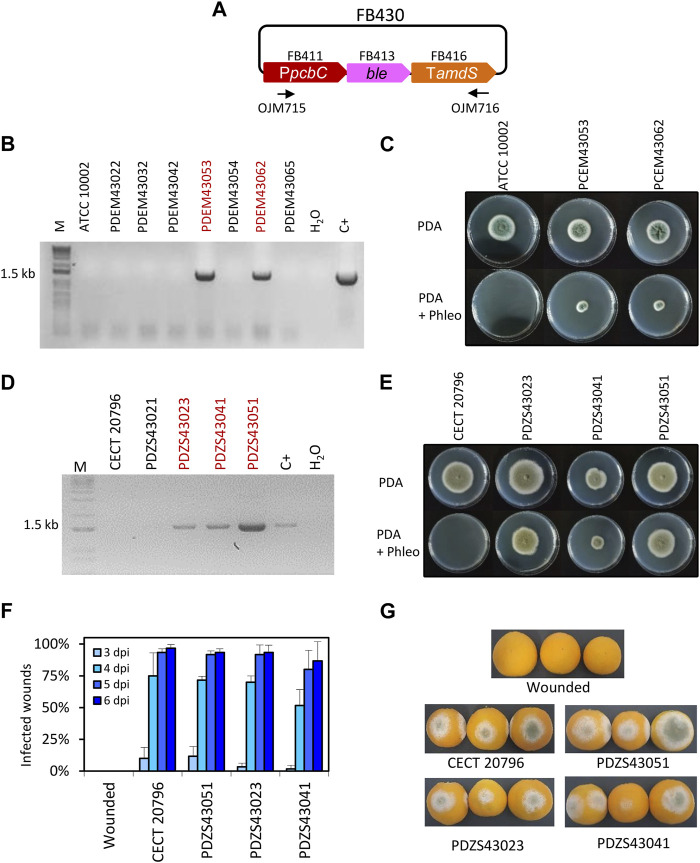
Functional validation of *ble* TU. **(A)** Plasmid pDGB3α2 FB430 for the ectopic integration of *ble* TU through ATMT to generate phleomycin resistance. Primers OJM715 and OJM716 were used for the molecular characterization of Phleo^R^ in *P. digitatum* and *P. chrysogenum* strains. **(B)** Molecular characterization of *P. chrysogenum* transformants. The 1.5 kb band corresponds to the complete *ble* TU. Selected strains are highlighted in red. **(C)** Growth profile of *P. chrysogenum* selected Phleo^R^ transformants after 7 days of growth in the presence of the antibiotic (25 μg/mL) at 25°C. **(D)** Molecular characterization of *P. digitatum* transformants. The 1.5 kb band corresponds to the complete *ble* TU as in **(B)**. Selected strains are highlighted in red. **(E)** Growth profile of *P. digitatum* Phleo^R^ transformants after 7 days of growth in the presence of the antibiotic (35 μg/mL) at 25°C. **(F)** Fruit infection assays of Phleo^R^ mutants on oranges. Data indicate the % of infected wounds (mean ± SD) at each day post-inoculation. No statistical difference was found between the parental CECT 20796 and the mutants at each dpi (*t*-test, *p* < 0.05). **(G)** Representative images of oranges infected by the indicated strains at 6 dpi.

**FIGURE 2:**
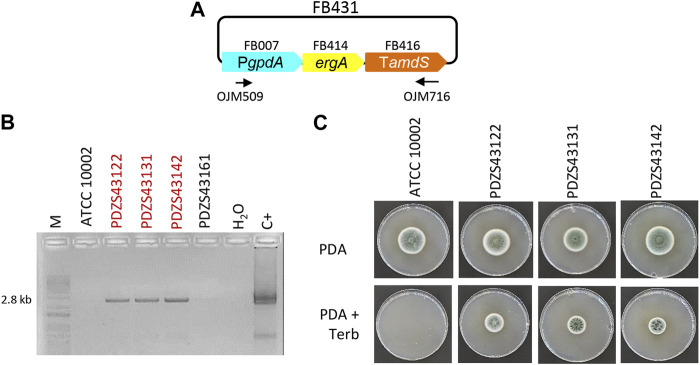
Functional validation of *ergA* TU in *P. chrysogenum*. **(A)** Plasmid pDGB3α2 FB431 for the ectopic integration of *ergA* TU through ATMT to generate terbinafine resistance. Primers OJM509 and OJM716 were used for the molecular characterization of the Terb^R^ strains shown in **(B)**. The 2.8 kb bands correspond to the complete *ergA* TU. Selected strains are highlighted in red. **(C)** Growth profile of selected Terb^R^ transformants after 7 days of growth in the presence of the antibiotic (0.5 μg/mL) at 25°C.

**FIGURE 3:**
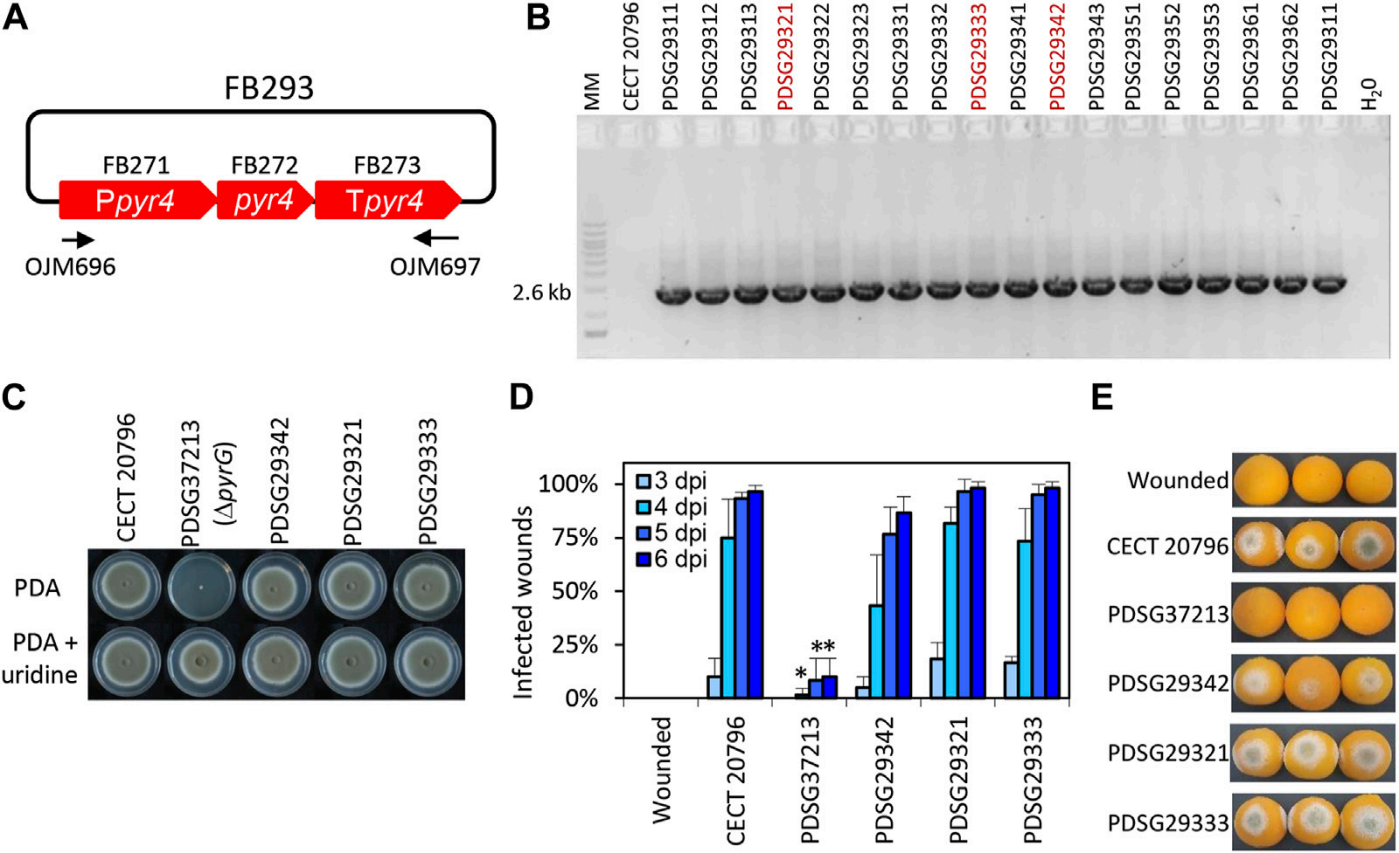
Functional validation of *T. reesei pyr4* TU in *P. digitatum*. **(A)** Plasmid pDGB3α2 FB293 for the ectopic integration of *pyr4* TU through ATMT to restore uridine auxotrophy. Primers OJM696 and OJM697 were used for the molecular characterization of the non-auxotrophic *P. digitatum* strains shown in **(B)**. The 2.6 kb bands correspond to the complete *pyr4* TU. Selected strains are highlighted in red. **(C)** Growth profile of selected Δ*pyrG:pyr4* transformants grown on PDA plates supplemented with 1.22 g/L uridine. It is to be noted that PDSG372013 was used as the parental strain for transformation with FB293. **(D)** Fruit infection assays of Δ*pyrG:pyr4* mutants on orange fruits. Data indicate the % of infected wounds (mean ± SD) at each day post-inoculation (dpi). (*) shows statistical significance between each sample compared to the control CECT 20796 at each dpi (*t*-test, *p* < 0.05). **(E)** Representative images of oranges infected by the indicated strains at 6 dpi.

All transformants were molecularly confirmed by PCR reactions using NZYTaq II DNA polymerase (NZYTech) (Figures 1–3; Supplementary Figures S1–S3) from genomic DNA isolated with the NZY Tissue gDNA isolation kit (NZYTech) and primers purchased from IDT (Table 2).

For the validation of GB_SynP promoters, *P. digitatum* protoplasts from strains carrying luciferase reporter vectors for each of the three synthetic promoters tested (FB398, FB399, and FB400) (Table 1) were transformed with the self-replicative AMA1-based dCas9-containing plasmid pAMA18.0_gRNA1 (Mózsik et al., 2021), as previously described (Garrigues et al., 2022). Transformants were selected on PDA plates containing 0.95 M sucrose and 35 μg/mL phleomycin. Regarding the reusability of the system, the loss of the pAMA18.0_gRNA1 plasmid was confirmed after three consecutive streaks of the transformants in non-selective PDA plates, as previously described (Garrigues et al., 2022).

### 2.4 Luciferase/nanoluciferase assays

*P. digitatum* and *P. chrysogenum* strains carrying the luciferase reporter vectors for each of the tested promoters (FB433, FB434, FB435, FB436, FB438, FB439, and FB441) and *P. digitatum* strains with the luciferase reporter for the GB_SynP synthetic promoters (FB398, FB399, and FB400), carrying or not pAMA18.0_gRNA1, were grown in duplicate for 2 days in 100 mL flasks with 25 mL of liquid potato dextrose broth (PDB, Difco-BD Diagnostics) at 25°C, with shaking (150 rpm). For induction assays, transformants were grown in duplicate for 4 days in 100 mL flasks with 25 mL of either *P. digitatum* or *P. chrysogenum* minimal medium (PdMM or PcMM, respectively) (Sonderegger et al., 2016) using 2% D-glucose (PanReac), 2% maltose (Sigma-Aldrich) or 2% D-xylose (Sigma-Aldrich) as the sole carbon source. Grown mycelia were filtered, and a sample of 20 mg was collected and immediately frozen in liquid nitrogen. Luciferase and nanoluciferase measurements were performed using the Dual-Glo^®^ Luciferase Assay System (Promega), as previously described (Gandía et al., 2022). Briefly, frozen samples were homogenized in 180 µL passive lysis buffer with a pestle and centrifuged (12,000 ×*g*, 10 min at 4°C). Then, 10 µL of the supernatant was transferred to a white 96-well plate (Thermo Fisher Scientific) and mixed with 40 µL of the luciferase reagent to measure luciferase luminescence in a CLARIOStar microplate reader (BMG LABTECH GmbH) with a measurement of 10 s and a delay of 2 s. Nanoluciferase luminescence was quantified thereafter by adding 40 µL of the Stop & Glo reagent and measured in the same way.

The luciferase/nanoluciferase ratio was determined for each sample, and normalized luminescence was calculated as the mean value of the ratios obtained from each duplicate. Statistical analyses were performed with GraphPad Prism 8.0.1 software. Differences between the strains were analyzed using one-way ANOVA, followed by *post hoc* multiple comparison Tukey’s test (*p* < 0.05). For induction experiments, we analyzed the differences in the growth of each strain in the presence of different carbon sources using Student’s *t*-test (*p* < 0.05).

### 2.5 Fruit infection assays

*P. digitatum* parental and mutant strains were inoculated on freshly harvested oranges (*Citrus sinensis* L. Osbeck cv Lane late), as previously described (González-Candelas et al., 2010). Briefly, three replicates of five orange fruits were inoculated with 5 μL of fungal conidial suspension (10^4^ conidia/mL) at four equidistant wounds around the equator. Control mock inoculations were performed with 5 μL of sterile Milli-Q H_2_O. Once inoculated, fruits were maintained at 20°C and 90% relative humidity for up to 6 days. Each inoculated wound was scored daily for infection symptoms on consecutive days post-inoculation (dpi). We repeated the experiments twice. Differences in the percentage of infection for each strain compared to the control CECT 20796 were analyzed using Student’s *t*-test (*p* < 0.05) for each individual dpi.

## 3 Results

### 3.1 Selection markers for antibiotic resistance

The FB platform already contains some commonly used positive fungal selection markers based on antibiotic resistance, such as *hph* (hygromycin^R^, FB003) or *nptII* (geneticin^R^, FB009) (Hernanz-Koers et al., 2018). However, in the case of integrative approaches, multiple genetic modifications often depend on the availability of different antibiotic resistance genes for transformant selection, which can be a bottleneck for the exploitation of filamentous fungi. In this study, we expand the range of selection markers available in the FB platform by including two alternative antibiotic resistance-inducing genes, the *ble* resistance gene from the bacterial transposon Tn5 and the squalene epoxidase *ergA* gene from *P. chrysogenum*. The expression of the *ble* gene provides the selection of the antibiotic phleomycin (Austin et al., 1990), whereas the expression of the *ergA* gene provides resistance against the antibiotic terbinafine in a broad range of filamentous fungi (Austin et al., 1990; Sigl et al., 2010).

In order to include *ble* resistance in the FB platform, a functional TU was generated. For this, we assembled the *ble* coding sequence (FB413), together with the promoter of isopenicillin N synthase (P*pcbC*) from *Penicillium rubens* (FB411) (Polli et al., 2016) and the terminator from the acetamidase (T*amds*) from *Aspergillus nidulans* (FB416) (Kelly and Hynes, 1985) into the pDGB3α2 vector to obtain FB430 (Table 2; Figure 1A) via restriction–ligation reactions. To functionally validate the resulting construct, we transformed *P. chrysogenum* and *P. digitatum* wild-type strains with the same FB430 via ATMT for the ectopic integration of the *ble* TU. *P. chrysogenum* transformants grown in the presence of 25 μg/mL phleomycin were selected and analyzed by PCR for the presence of the *ble* cassette (Figure 1B). The positive transformants, PCEM43053 and PCEM43062, showed growth on phleomycin-containing plates when compared to the parental ATCC 10002 (Figure 1C), further demonstrating the functionality of FB430. In parallel, FB430 was also validated in *P. digitatum* (Figures 1D–G). *P. digitatum* transformants grown in the presence of 35 μg/mL phleomycin were selected and confirmed by PCR (Figure 1D). The positive transformants PDZS43023, PDZS43041, and PDZS43051 were able to grow on phleomycin-containing plates (Figure 1E) and showed the same pathogenicity as the parental CECT 20796 in orange fruits (Figures 1F,G).

Similarly, to incorporate the terbinafine resistance-inducing gene in the FB platform, a functional TU for *ergA* was generated and validated in *P. chrysogenum* (Figure 2). We assembled the *ergA* coding sequence (FB414), together with the P*gpdA* promoter (FB007) and the T*amds* terminator (FB416), into the pDGB3α2 vector to obtain the FB431 construct (Table 2; Figure 2A). *P. chrysogenum* transformants grown on 0.5 μg/mL terbinafine were chosen and confirmed by PCR (Figure 2B). The positive transformants PDZS43122, PDZS43131, and PDZS43142 could grow on phleomycin-containing PDA plates in contrast to the parental ATCC 10002 (Figure 2C), demonstrating the functionality of FB431.

Overall, both resistances were transformed ectopically to avoid any bias regarding the targeting of specific loci, and these experiments validated the use of FB430 and FB431 as standardized TUs for conferring positive selection in the transformation of different fungal species, expanding the antibiotic resistance selection markers currently available in the FB system.

### 3.2 Selection markers based on fungal auxotrophy

To date, no auxotrophic markers have been included in the FB platform despite the fact that they are sustainable alternatives for the use of antibiotics in transformant selection. The orotidine 5′-phosphate decarboxylase *pyr4* gene from *T. reesei* is widely used as an auxotrophic selection marker that can be counter-selected using 5-FOA or fully supplemented using uridine (Díez et al., 1987; Derntl and Kiesenhofer, 2015). In this study, we set up several experiments to design, test, and validate *pyr4* as a selection marker in *pyr4/pyrG-*deficient fungal strains. As a first step, uridine-auxotrophic *P. digitatum* Δ*pyrG* mutants were generated through ATMT using FB372 as the template for homologous recombination at the *pyrG* locus (Table 2; Supplementary Figure S4). Transformants were selected on PDA plates supplemented with 1.22 g/L uridine and 1.25 g/L 5-FOA, and were molecularly and phenotypically characterized (Supplementary Figure S4). Growth profiles showed that after *pyrG* deletion, *P. digitatum* mutants could no longer grow on PDA plates unless supplemented with uridine, confirming their auxotrophic condition. Additionally, these mutants could also grow in the presence of uridine and 5-FOA in contrast to the parental CECT 20796, further confirming *pyrG* deletion. Finally, infection assays on orange fruits revealed that *P. digitatum* Δ*pyrG* mutants showed highly reduced pathogenesis compared to the control (Supplementary Figure S4). Once the *pyrG* deletion mutants were obtained, a functional TU for the *T. reesei pyr4* gene was generated. For this, we assembled the *Penicillium* codon-optimized and extensively domesticated *pyr4* coding sequence (FB272), promoter (FB271), and terminator (FB276) into the pDGB3α2 vector to obtain FB293 (Table 2; Figure 3A) via restriction–ligation reactions. To functionally validate the resulting construct FB293, we transformed the *P. digitatum* Δ*pyrG* mutant PDSG37213 with FB293 via ATMT for the ectopic integration of *pyr4* TU. Transformants grown on PDA plates were assessed by PCR (Figure 3B) and phenotypically analyzed to confirm *pyrG:pyr4* complementation and, therefore, the absence of the auxotrophy. As shown in Figure 3C, complemented mutants PDSG29312, PDSG29321, and PDSG29333 were all able to grow on PDA plates without uridine, in contrast to the auxotrophic parental PDSG37213. Remarkably, *P. digitatum* complemented mutants that fully recovered their original pathogenicity (Figures 3D,E), which validates *pyr4* as an auxotrophic selectable marker also for (phyto)pathogenic fungi, in which the deletion of *pyrG* orthologs has been demonstrated to decrease pathogenicity and virulence in the corresponding fungi (Zameitat et al., 2007; Higashimura et al., 2022).

### 3.3 Constitutive and inducible promoters

**FIGURE 4:**
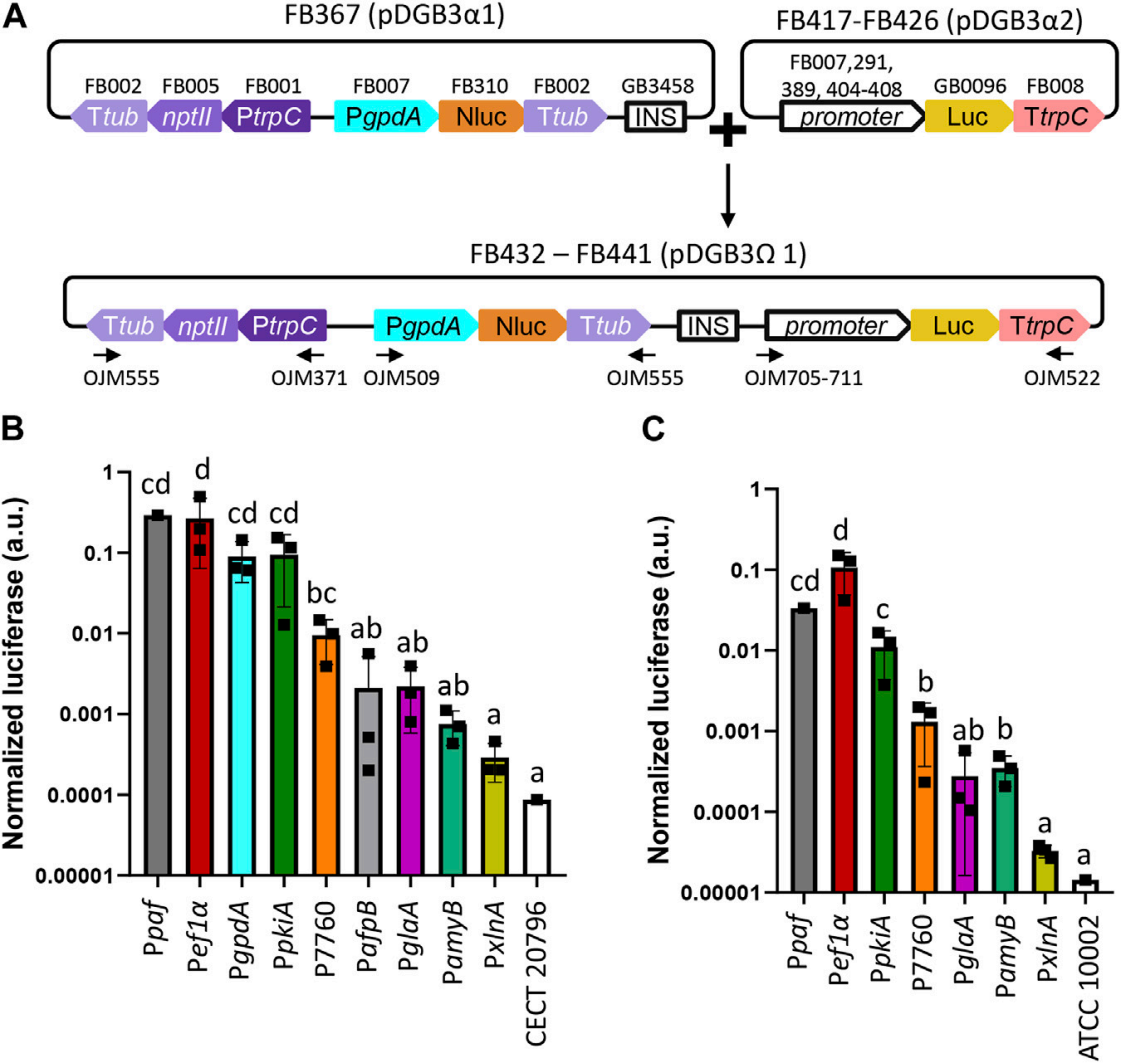
Functional promoter validation via the luciferase assay in *P. digitatum* and *P. chrysogenum*. **(A)** Scheme of the assembly architecture used to express the luciferase reporter system. Different promoters were tested using luciferase as a reporter, and the constitutive expression of nanoluciferase under the P*gpdA* promoter was used as a reference for normalization. All constructs included a geneticin resistance gene (*nptII*) for the selection of positive transformants. An insulator sequence was introduced between the nanoluciferase and luciferase genes to allow the binary assembly of plasmids. Primers used for the molecular characterization of transformants are indicated with arrows. **(B)** Normalized luciferase expression for each promoter in *P. digitatum* transformants grown in PBD for 2 days. **(C)** Normalized luciferase expression for each promoter in *P. chrysogenum* transformants grown in PBD for 2 days. Constitutive expression of luciferase under the P*paf* promoter was included as a reference. Letters denote statistical significance between values in a one-way ANOVA (Tukey’s multiple comparisons test, *p* ≤ 0.05). Error bars represent the average values ±SD (n = 9). Squares represent the mean value of each of the three biological replicates (transformants) measured twice. It is to be noted that the Y-axis is represented in the logarithmic scale.

In order to further expand and characterize the promoter catalog available in the FB platform, a series of promoters from different fungal species were included in the collection (Table 1) and were functionally validated using a luciferase reporter system, as previously described (Gandía et al., 2022). This reporter consists of two TUs: the nanoluciferase coding sequence (Nluc, FB310) under the regulation of the P*gpdA* promoter that serves as an internal standard for normalization, and the luciferase sequence (Luc, GB0096) under the regulation of the promoter to be tested. Promoter strength is expressed as the ratio of the Luc signal divided by the Nluc internal standard. The promoters to be evaluated included the previously characterized strong pyruvate kinase gene promoter (P*pkiA*) from *Aspergillus niger* (FB389) (de Graaff et al., 1992). Novel promoters from *P. digitatum* included the antifungal protein AfpB gene promoter P*afpB* (FB404) (Garrigues et al., 2017), and two promoters with high expression levels were reported in a previous transcriptomic study: the elongation factor 1α gene promoter (P*ef1*α) (FB407) and the ubiquitin ligase PDIG_07760 gene promoter (P07760) (FB408) (Ropero-Pérez et al., 2023). The inducible promoters included in this study are the endo-1,4-β-xylanase A gene promoter (P*xlnA*) from *A. nidulans* (FB291, xylose-responsive) (Orejas et al., 1999), the glucoamylase gene promoter (P*glaA*) from *A. niger*, and the TAKA-amylase A gene promoter (P*amyB*) from *Aspergillus oryzae* (FB405 and FB406, respectively, both maltose/starch-responsive) (Fowler et al., 1990; Tsuchiya et al., 1992b). The widely used glyceraldehyde-3-phosphate dehydrogenase promoter P*gpdA* from *A. nidulans*, which was already available in the FB collection (FB007) (Hernanz-Koers et al., 2018), was also included in the analysis, as well as the luciferase reporter construct for the *P. chrysogenum* antifungal protein PAF promoter (P*paf*) from previously published data (FB323) (Gandía et al., 2022) to serve as references. To facilitate the cloning of new luciferase reporter constructs, the nanoluciferase reference gene and the *nptII* resistance gene were cloned into a pDGB3α1 vector (FB367) to be combined in a single reaction with the luciferase TUs cloned into pDGB3α2 vectors that included the promoters to be tested (Figure 4A). Due to the requirements of the FB binary assembly, an insulator sequence (GB3458) was also included at the 3’ end of the FB367 vector, which also helps prevent the interaction between Nluc and Luc TUs. The luciferase reporter constructs for each of the assayed promoters (FB432 to FB441) showed different normalized luciferase expression levels in *P. digitatum* after 2 days of growth in PDB (Figure 4B). The lowest expression levels were observed for the inducible promoters P*xlnA*, P*amyB*, and P*glaA*, in this order, due to the lack of inducers in this medium. Their expression levels, together with that driven by P*afpB,* were slightly above the basal signal observed in the control strain, but these were not statistically significant. The expression driven by P*pkiA*, P*gpdA*, and the new P*ef1*α were similar to those observed for P*paf*, while P07760 showed intermediate expression values between these expression and the inducible promoters.

The selected constructs carrying P*xlnA*, P*pkiA*, P*glaA*, P*amyB*, P*ef1*α, and P07760 were also transformed into *P. chrysogenum*, in which the luciferase expression level was about 10 times lower than the overall levels observed in *P. digitatum* (Figure 4C), except for P*ef1*α and P*amyB*, which showed a similar signal in both fungal chassis (0.27 and 0.1 for P*ef1*α in *P. digitatum* and *P. chrysogenum*, respectively, and 0.0007 and 0.0004 for P*amyB*). Unlike *P. digitatum*, the signal driven by P*amyB* was above the basal signal in *P. chrysogenum* and showed similar expression levels to that of the P7760 promoter. Relative expression levels among the other promoters were nevertheless maintained in both fungi, with P*pkiA* and P*ef1*α signals similar to that of the P*paf* reference promoter and with P*xlnA-*, P*glaA-*, and P*amyB*-driven signals similar to that of the control strains.

### 3.4 Induction of P*glaA*, P*amyB*, and P*xlnA* promoters

**FIGURE 5:**
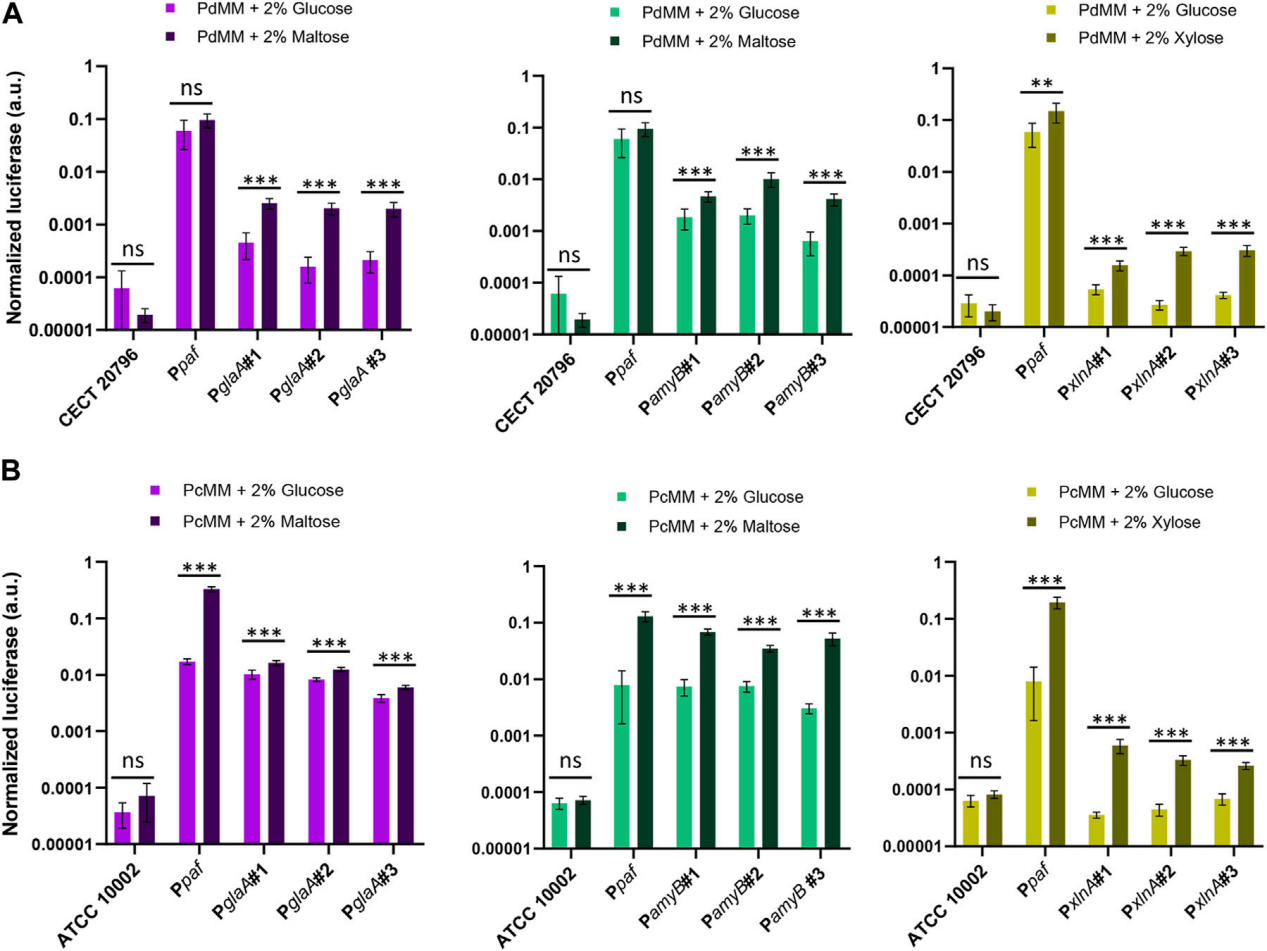
Activation of inducible promoters P*glaA*, P*amyB*, and P*xlnA* in *P. digitatum* **(A)** and *P. chrysogenum* **(B)**. Expression was measured after 4 days of growth in minimal medium, replacing glucose with maltose as the carbon source for P*amyB* and P*glaA* transformants, and with xylose for P*xlnA* transformants. The expression of luciferase under the P*paf* promoter was also included as a reference. Asterisks represent statistical significance (Student’s *t*-test, ns = *p* ≥ 0.05, *p < 0.05, **p < 0.01, and ***p < 0.001) between the expression levels of each individual transformant in MM with maltose/xylose and those observed in the reference MM with glucose. Error bars represent the average values ±SD (n = 6). It is to be noted that the Y-axis is represented in the logarithmic scale.

In order to further characterize the inducible promoters included in this study, we analyzed the induction of luciferase expression directed by P*glaA*, P*amyB,* and P*xlnA* promoters in *P. digitatum* (Figure 5A) and *P. chrysogenum* strains (Figure 5B) after 4 days of growth in minimal medium (PdMM for *P. digitatum* and PcMM for *P. chrysogenum*) using different inducers as the sole carbon source (2% maltose for P*glaA* and P*amyB*, and 2% xylose for P*xlnA*). When the fungi were grown in the presence of the inducer, the expression levels driven by all three promoters were significantly higher than those observed in the reference media with 2% glucose, which increase by 8x, 4x, and 10x for P*glaA*, P*amyB*, and P*xlnA*, respectively, in *P. digitatum* (Figure 5A); and by 2x, 9x, and 8x in *P. chrysogenum* (Figure 5B). Signals observed for the P*xlnA* promoter in the reference media (MM + glucose) were similar to the basal signal of the reference strains in both fungi, while P*amyB* and P*glaA* promoters showed higher basal expression in the same media, especially in *P. chrysogenum.* Remarkably, expression levels in the reference P*paf* promoter were found to increase significantly in *P. chrysogenum* when grown in the PcMM supplemented with maltose or xylose (0.22 a.u. on average) when compared to the medium supplemented with glucose (0.015 a.u. on average, 15 times lower). The same was observed, but to a lesser extent, in *P. digitatum* when P*paf* was expressed in PdMM glucose (average values of 0.6 a.u.) compared to its expression in PdMM xylose (0.15 a.u. average values, four times higher).

### 3.5 dCas9-activated synthetic promoters

**FIGURE 6:**
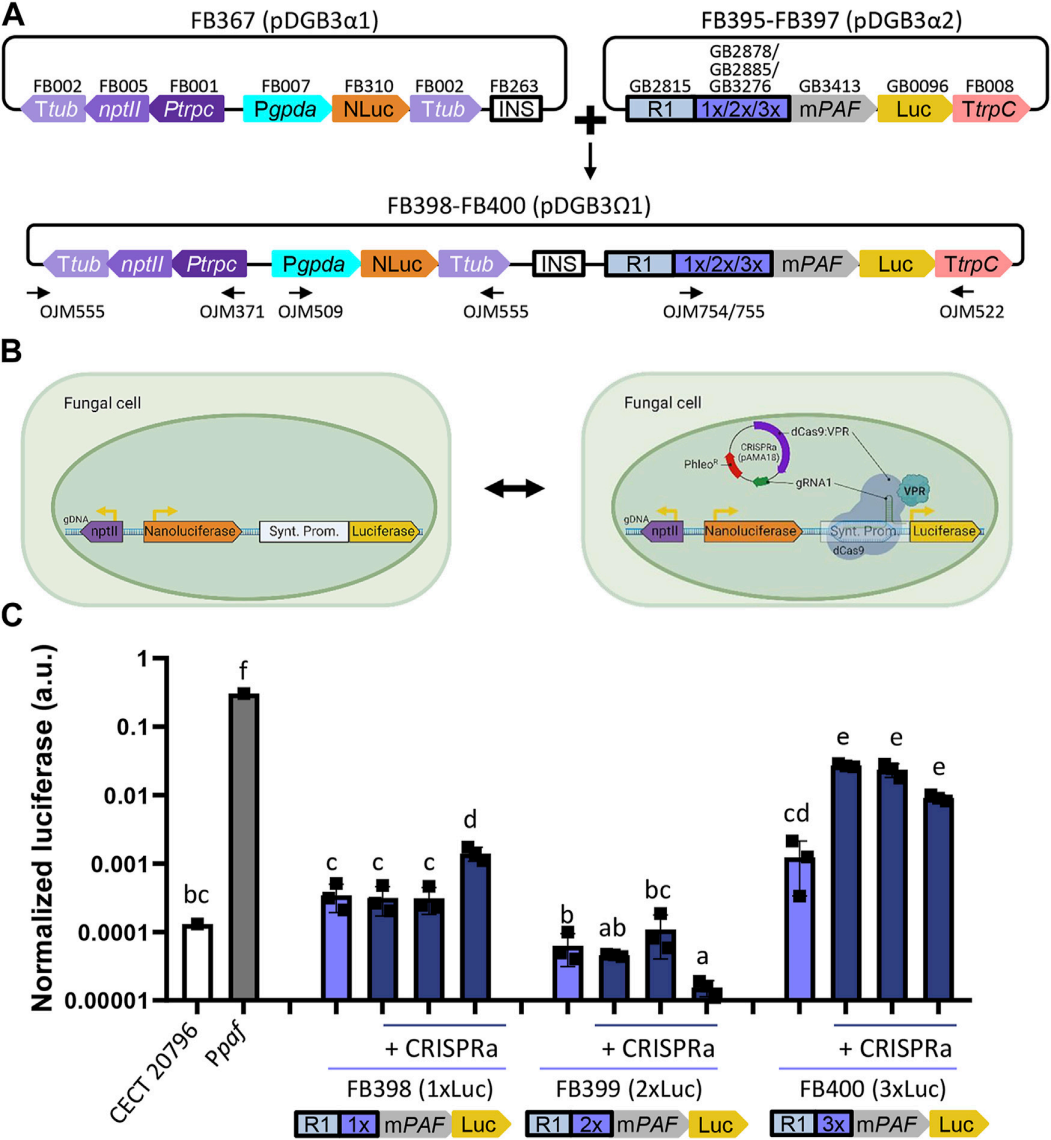
Functional validation of GB_SynP in *P. digitatum*.** (A)** Scheme of the construct architecture used to constitutively express the luciferase reporter system using the dCas9-regulated synthetic promoters with low (1xLuc, FB398), medium (2xLuc, FB399), or high (3xLuc, FB400) promoter strength. Constructs included the constitutive expression of nanoluciferase under the P*gpdA* promoter as a reference for normalization, geneticin resistance for the selection of the transformants, and an insulator sequence to allow the binary assembly of plasmids. Oligos used for the molecular characterization of transformants are indicated with arrows. **(B)** Schematic representation of the dCas9-activated luciferase reporter system. Expression of geneticin resistance and nanoluciferase is constant, while the expression of luciferase is only achieved in the presence of the dCas9-based activation system contained in the pAMA18-derived plasmid. **(C)** Expression of positive transformants for FB398 (1xLuc), FB399 (2xLuc), or FB400 (3xLuc) in the presence (+CRISPRa) or absence of the pAMA18.0_gRNA1 plasmid. Constitutive expression of luciferase under the P*paf* promoter was included as a reference. Squares represent the mean value of each of the three biological replicates (transformants) measured twice. Letters denote statistical significance between values in a one-way ANOVA (Tukey’s multiple comparison test, *p* ≤ 0.05). Error bars represent the average values ±SD (n = 6). It is to be noted that the y-axis is represented in the logarithmic scale. Figure includes images created with BioRender (biorender.com).

Finally, we tested the recently developed GB_SynP (Moreno-Giménez et al., 2022) in our fungal chassis in combination with the pAMA18.0_gRNA1 plasmid, which delivers the CRISPRa system necessary to activate GB_SynP promoters in a non-integrative manner (Mózsik et al., 2021). To this end, we developed luciferase reporter constructs following the same procedure as for natural promoters (Figure 6A). In these constructs, luciferase expression was regulated by synthetic promoters consisting of an A1 distal promoter part formed by a random sequence (GB2815), and an A2 proximal promoter part including the target sequence for gRNA1 that was repeated once (GB2878), twice (GB2885), or three times (GB3276), and the minimal promoter m*PAF* (GB3423) derived from the native P*paf* from the fungus *P. chrysogenum*, which was previously found to drive a strong induction when exposed to the dCas9 system loaded with gRNA1 in plants (Moreno-Giménez et al., 2022). The resulting constructs containing one gRNA1 target (FB395, 1xLuc), two targets (FB396, 2xLuc), or three targets (FB397, 3xLuc) were stably transformed into *P. digitatum* via ATMT. Protoplasts obtained from these strains were re-transformed with the CRISPRa expression vector pAMA18.0_gRNA1 (Figure 6B). The expression levels of 1xLuc and 2xLuc constructs were not significantly higher than the basal signal of the control strain despite the presence of pAMA18.0_gRNA1, except for one of the 1xLuc re-transformants, which showed a low, but statistically significant increase in the luciferase expression when compared to the same strain in the absence of the CRISPRa system. A higher and significant increase of approximately 16 times on average in the luciferase signal was observed for the 3xLuc construct (Figure 6C) in all tested re-transformants. Although the expression driven by this promoter was approximately 10 times lower than that observed with the reference P*paf* promoter*,* it is comparable to that achieved with P*glaA* and P*amyB* **,** showing the functionality of these dCas9-activated synthetic promoters in fungi.

## 4 Discussion

The FB cloning platform allows for the open exchange of standardized, ready-to-use DNA parts in the fungal research community (Hernanz-Koers et al., 2018). Moreover, if the platform is functionally compatible and validated, it also allows for the exchange of parts between plants and fungi, as occurred with the fluorescent YFP protein or the hygromycin selection marker reported previously (Hernanz-Koers et al., 2018) or the synthetic promoters reported in this study. However, the number of validated genetic elements present in the FB platform was very limited to date, which hindered the biotechnological exploitation of filamentous fungi. In this study, we have expanded the available genetic elements in FB platforms by incorporating one auxotrophic selection marker (*pyr4*)*,* two additional antibiotic resistance markers (*ergA* and *ble*), two strong promoters (P*pkiA* and P*ef1α*), two intermediate promoters (P*afpB* and P7760), three inducible promoters (P*glaA*, P*amyB*, and P*xlnA*), and three versions of the dCas9-regulated GB_SynP synthetic promoters. Even though the validation of these new parts has been performed in *Penicillium* species, the FB system has been demonstrated to mediate the expression of the same construct in different fungal genera. For instance, the FB027 construct used for the expression of YFP has been functionally validated in *P. digitatum*, *Penicillium expansum*, and *A. niger* (Hernanz-Koers et al., 2018; Vazquez-Vilar et al., 2020). Therefore, the FB system and, subsequently, the new FB parts described here, are expected to be of use in a wide range of fungal species of different genera.

Since the FB release, there have been an increasing number of SynBio-based genetic toolkits developed for filamentous fungi (Dahlmann et al., 2021; Mózsik et al., 2021; Mózsik et al., 2022). In this sense, FB, which derives from the GB cloning framework, shares most of the codes and type IIS restriction enzymes with these alternative SynBio collections (Weber et al., 2011), making it possible to combine code-compatible level 0 plasmids between these systems to assemble TUs into level 1 plasmids. However, these Golden Gate-based collections alternative to FB use plasmids derived from pAMA1 or pEHN8, which are introduced into fungal cells via protoplast transformation. In contrast, the FB collection is based on pCAMBIA-derived vectors and can be applied to a broad spectrum of fungal species that are compatible with ATMT (de Groot et al., 1998), which is considered to be a more advantageous transformation method than protoplasts as spores can be used directly for genetic transformation and transformation efficiencies are generally higher (Li et al., 2017 and references, therein). Moreover, unlike these other Golden Gate cloning systems, FB/GB systems allow for the indefinite expansion of multigenetic constructs via bipartite assemblies between pDG3α and pDGB3Ω vectors (Sarrion-Perdigones et al., 2011). To date, the GB system has permitted the assembly of up to 10 TUs (GB3243) (Selma et al., 2022) and inserted an assembly as large as 20 kb (GB4559–GB4585) (Moreno-Giménez et al., 2022), yet the transformation and propagation of larger constructs into *E. coli* might be hampered by the limitations to this host (Weber et al., 2011). In this regard, the adaptation of other ATMT-compatible vectors into FB/GB could be considered, such as the binary-BAC (BIBAC) vector reported by Hamilton (1997), which can carry >100 kb and has already been used to transform *Fusarium, Aspergillus*, or *Ustilago* species (Takken et al., 2004; Ali and Bakkeren, 2011).

Among the new genetic elements in the FB system, we included three commonly used fungal selection markers, two of them based on antibiotic resistance (*ble* and *ergA*, which confer resistance to phleomycin and terbinafine, respectively) and one based on fungal auxotrophy (*pyr4*), further expanding the possibilities for fungal transformation and mutant selection within the frame of the FB platform. Although antibiotic resistance markers are among the most widely used approaches for positive transformant selection, auxotrophic markers are more sustainable alternatives for the use of antibiotics, which can have undesired side effects on the fitness of the organism under study or cause unwanted spontaneous resistance. The orotidine 5′-phosphate decarboxylase-encoding gene *pyr4* from *T. reesei*, which is an ortholog of the *Aspergillus* and *Penicillium pyrA*/*pyrG* gene, is widely applied as a strong auxotrophic selection marker that can be counter-selected using 5-FOA or fully supplemented using uracil or uridine (Díez et al., 1987; Derntl and Kiesenhofer, 2015). Interchangeability of these two orthologs has already been demonstrated between fungi from different phylogenetic classes, from Sordariomycetes to Ascomycetes and *vice versa* (Ballance and Turner, 1985; Díez et al., 1987; Gruber et al., 1990). Therefore, the FB-adapted *pyr4* TU is expected to restore uridine/uracil auxotrophy in a broad range of fungal species. In the case of fungal (plant) pathogens, for which *pyr* disruption has been reported to reduce pathogenicity and virulence (Zameitat et al., 2007; Higashimura et al., 2022), and as also demonstrated here for *P. digitatum* Δ*pyrG* for the first time (Supplementary Figure S4), *pyr4* complementation completely restored pathogenicity, thus demonstrating the suitability of this genetic element as an auxotrophic selectable marker, as well as for (phyto)pathogenic fungi.

The luciferase reporter system has allowed us to functionally validate and characterize seven distinct promoters that have been incorporated as standard DNA parts to the FB toolbox. Among these are novel promoter sequences for which their functionality had never been validated before (P*afpB*, P7760, and P*ef1*α). These promoters were selected either for their interesting behavior in a *P. digitatum-*based transcriptome analysis (Ropero-Pérez et al., 2023) or because they are well-known promoters, such as the strong P*pkiA* or the maltose-responsive P*glaA* and *PamyB*, all of which have been extensively used in *Aspergillus* species (Storms et al., 2005; Oliveira et al., 2008; Song et al., 2018). Among the inducible promoters included in this study, P*glaA* and P*xlnA* have already been implemented in alternative Golden Gate-compatible collections (Polli et al., 2016; Mózsik et al., 2021), yet the validation of either basal or induced states has not been described in *Penicillium* species. Herein, a wide expression range was found for all tested promoters, from the highly expressed P*pkiA* and P*ef1*α promoters, with levels similar to those of the well-known P*paf* and P*gpdA,* to lower- or almost no-expressed promoters, such as P07760 and P*afpB*. The expression levels of almost all these promoters were reduced in *P. chrysogenum* compared to *P. digitatum*, except for the newly characterized P*ef1*α promoter from *P digitatum*, which showed similar values in both fungal backgrounds. This likely reflects a greater orthogonality in this promoter, which may be of preferable use to ensure strong expression in other fungal chassis. Inducibility of P*glaA*, P*amyB*, and P*xlnA* was also validated using the luciferase reporter system in both *Penicillium* species, showing different expression ranges both in the presence and absence of the inducer. This allows for multiple options for the custom design of future experiments, allowing promoters with lower background expression to choose, such as P*xlnA*, when basal expression needs to be almost completely avoided or to prioritize activation over background expression with promoters such as P*amyB*. Interestingly, P*amyB* induction in *P. chrysogenum* was similar to that shown in the industrial workhorse *A. oryzae* using β-glucuronidase (GUS) as the reporter. The expression of this promoter was reported to increase 10 times in a maltose-containing medium compared to glucose (Ozeki et al., 1996). This expression was slightly higher (1250 U/mg) (Tada et al., 1991) than that reported for P*glaA* (903 mg U/mg) (Hata et al., 1992), which correlates with our results shown in Figure 5B. Both P*glaA* and P*amyB* promoters are commonly applied for the production of different proteins of interest, such as human tissue plasminogen (Wiebe et al., 2001), bovine chymosin (Ohno et al., 2011), or synthetic human lysozyme (Tsuchiya et al., 1992a), which further shows their relevance in the field of fungal biotechnology. On the other hand, the use of the P*xlnA* promoter is very limited to date, being its ortholog, P*xylP* from *P. chrysogenum* more extensively used (Yap et al., 2022). Herein, we demonstrate the possibility of implementing this promoter in the *Penicillium* genus, with more modest induction levels than those of P*glaA* and P*amyB* but with the lack of basal expression in the absence of the inducer.

Unexpectedly, the P*paf* expression was found to significantly increase in the presence of maltose in *P. chrysogenum* and xylose in both *P. chrysogenum* and *P. digitatum.* This would suggest that (i) maltose and xylose themselves or any of the maltose/xylose catabolic intermediates serve as inducers for P*paf* or (ii) P*paf* expression is partially repressed by glucose, which can be attributed to the presence of carbon catabolite repression CREA motifs in the P*paf* sequence, as previously described (Marx et al., 1995). This repression is nevertheless almost completely lost when P*paf* is expressed in a different fungal chassis, such as *P. digitatum*, suggesting different regulatory mechanisms between both fungal species despite their phylogenetic proximity.

The activation of GB_SynP promoters in *P. digitatum* was addressed using the luciferase reporter system and the CRISPRa system included in the pAMA18.0_gRNA1 vector (Mózsik et al., 2021). The non-integrative nature of this pAMA1-based plasmid makes it possible to revert promoter activation upon plasmid loss in the absence of selection pressure (Garrigues et al., 2022). Additionally, this CRISPRa system provides a method to easily assay expression variations within the same background strain, either by testing different activation domains or inducible systems, or by analyzing the induction level under different culture conditions. The activation of 1xLuc and 2xLuc constructs, however, was not achieved in *P. digitatum* using the pAMA18.0_gRNA1 vector as signals of all but one 1xLuc re-transformant were on the same range as the basal signal of the reference strain. The expression of 1xLuc was, however, not different from the expression observed in 3xLuc strains in the absence of the CRISPRa system, which could mean that this expression is within the range of basal expression of the synthetic promoters. On the other hand, in the case of the 3xLuc construct, we did observe an increase of more than one order of magnitude in the presence of the CRISPRa system compared to the non-activation control for all 3xLuc re-transformants tested. These results indicate that the activation of these promoters in fungi requires the presence of at least three repetitions of the gRNA target, which highly differ from what was observed in plants, where one repetition of the target sequence for gRNA1 was sufficient to drive a significant increase of synthetic promoter expression (Moreno-Giménez et al., 2022). The discrepancies in the GB_SynP behavior between plants and fungi could be attributed to the differences in CRISPRa systems used in each organism. Although pAMA18.0_gRNA1 used for activation in *P. digitatum* comprised the dCas9 protein fused to the VPR activation domain, the dCasEV2.1 complex used for activation in *Nicotiana benthamiana* plants includes a dCas9 protein fused to an EDLL activation domain and an extra MS2 protein fused to the VPR domain that is able to recognize and bind the modified gRNA scaffold (Selma et al., 2019; Moreno-Giménez et al., 2022). Although VPR showed a major contribution in the activation as the expression levels dropped significantly when MS2 was fused to other activation domains (Moreno-Giménez et al., 2022), in fungi, a second activation component might be required to reach higher activation levels. Another explanation for low expression levels in fungi may reside in gRNA1 used to trigger the activation of GB_SynP promoters, which was originally designed for plants. Although no off-targets were found for gRNA1 in the *P. digitatum* CECT 20796 genome, the efficiency of this gRNA may not be optimal for this chassis, and therefore, a gRNA designed specifically for fungi might enhance the activated expression levels. Additionally, expression levels in fungi could also be enhanced by creating new A2 proximal promoter parts with more than three repetitions of the gRNA1 target sequence. Further optimization of GB_SynP promoters for filamentous fungi, following these guidelines, will be explored in the near future to better characterize this tool and its potential for wide-range expression of customizable synthetic promoters in filamentous fungi.

## 5 Conclusion

FungalBraid 2.0 aims to accelerate the development of fungal SynBio by the inclusion of a new repertoire of 27 domesticated DNA parts, resulting in functionally validated resistance and auxotrophic markers, as well as strong, inducible, and synthetic promoters for their contributory use by the fungal research community. With the newly expanded FB toolkit, a greater number of modular DNA assemblies are possible, exponentially increasing the possibilities for the study, development, and exploitation of filamentous fungi as cell biofactories.

## Data Availability

The datasets presented in this study can be found in the online repository DIGITAL CSIC (https://digital.csic.es/handle/10261/308066). DNA parts generated in this work can be found at: https://gbcloning.upv.es/ and https://www.addgene.org/ accession number(s) can be found in the Supplementary Material.
