# Supplementary material for: FungalBraid 2.0: expanding the synthetic biology toolbox for the biotechnological exploitation of filamentous fungi
Source: Front Bioeng Biotechnol. 2023 Aug 7;11:1222812. doi: 10.3389/fbioe.2023.1222812 (PMC10441238; doi:10.3389/fbioe.2023.1222812)
Supplement: Supplementary file 1 [file DataSheet1.pdf]

Supplemental material

FungalBraid 2.0: expanding the synthetic biology toolbox for the biotechnological exploitation of filamentous fungi

Elena Moreno-Giménez[1,2], Mónica Gandía[3], Zara Sáez[1], Paloma Manzanares[1], Lynne Yenush[2], Diego Orzáez[2], Jose F. Marcos[1]* and Sandra Garrigues[1]*

[1] Food Biotechnology Department, Instituto de Agroquímica y Tecnología de Alimentos (IATA), Consejo Superior de Investigaciones Científicas (CSIC), Catedrático Agustín Escardino Benlloch 7, 46980 Paterna, Valencia, Spain.

[2] Instituto de Biología Molecular y Celular de Plantas (IBMCP), Consejo Superior de Investigaciones Científicas (CSIC)-Universitat Politècnica de València (UPV), Valencia 46022, Spain.

[3] Preventive Medicine and Public Health, Food Science, Toxicology and Forensic Medicine Department. Faculty of Pharmacy. Universitat de València. Vicente Andrés Estellés s/n, Burjassot 46100, Valencia, Spain.

*Corresponding authors: Jose F. Marcos (jmarcos@iata.csic.es) and Sandra Garrigues (sgarrigues@iata.csic.es)

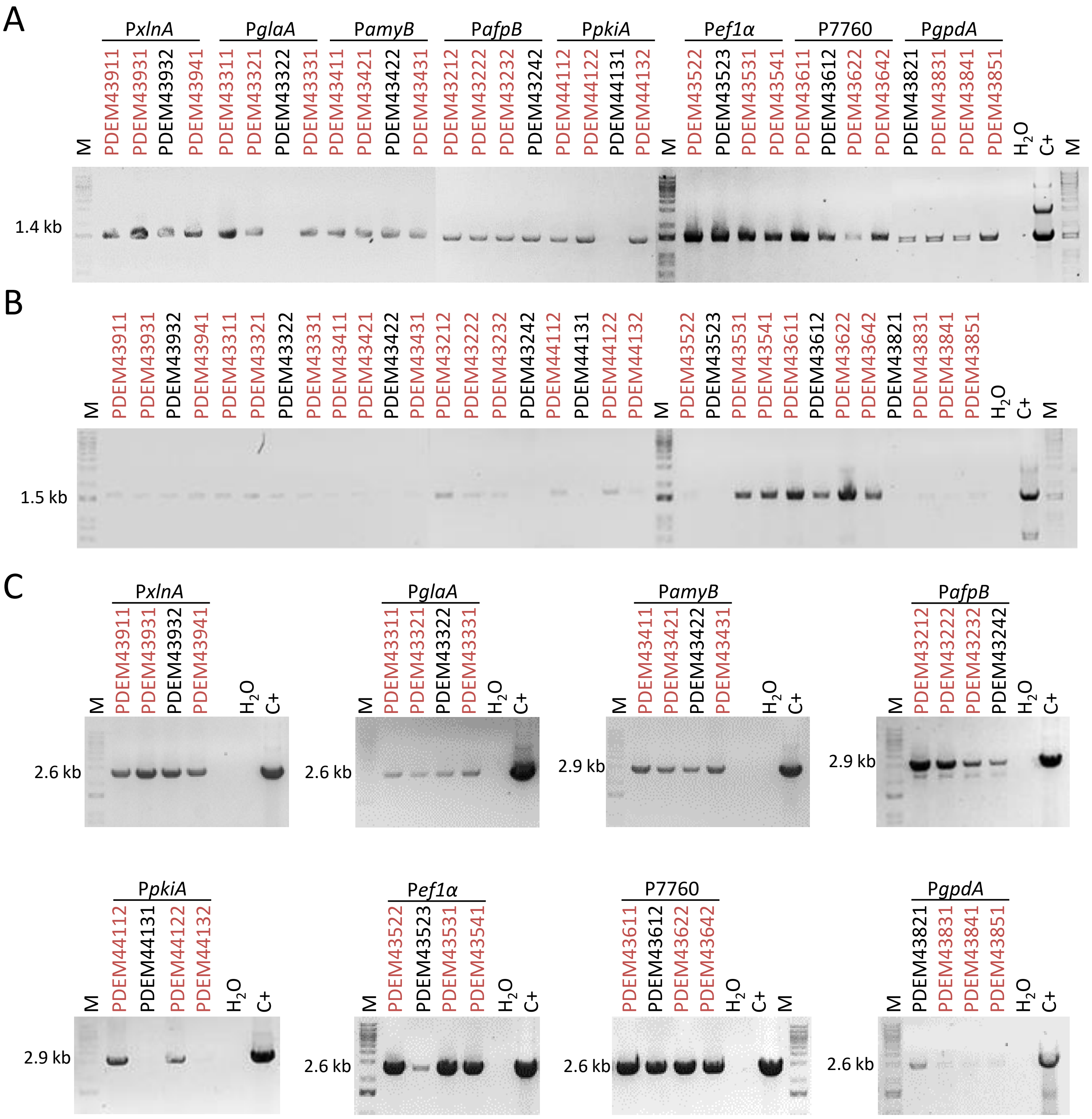

Supp. Figure S1. Molecular characterization of *P. digitatum* strains transformed with luciferase reporter system for each of the assayed promoters. (A) Amplification of the *nptII* geneticin resistance TU (1.4 kb) using primers OJM371 and OJM555. (B) Amplification of Nanoluciferase TU (1.5 kb) using primers OJM509 and OJM555. (C) Amplification of luciferase TUs for each construct using the reverse primer OJM522 and the corresponding forward primer for each promoter (OJM509 for P*gpdA*, OJM705 for P*xlnA*, OJM706 for P*pkiA*, OJM707 for P*afpB*, OJM708 for P*glaA*, OJM709 for P*amyB*, OJM710 for P*ef1A*, and OJM711 for P7760). The 2.6 kb bands correspond to the complete luciferase TU when expressed under the promoter P*xlnA*, P*glaA*, P*gpdA*, P7760 or P*ef1A*, and the 2.9 kb bands correspond to the luciferase TU under P*amyB*, P*kiA* or P*afpB*. A total of 4 transformants for each construct were analyzed. Names in red correspond to the validated transformants that were selected for the luciferase assay.

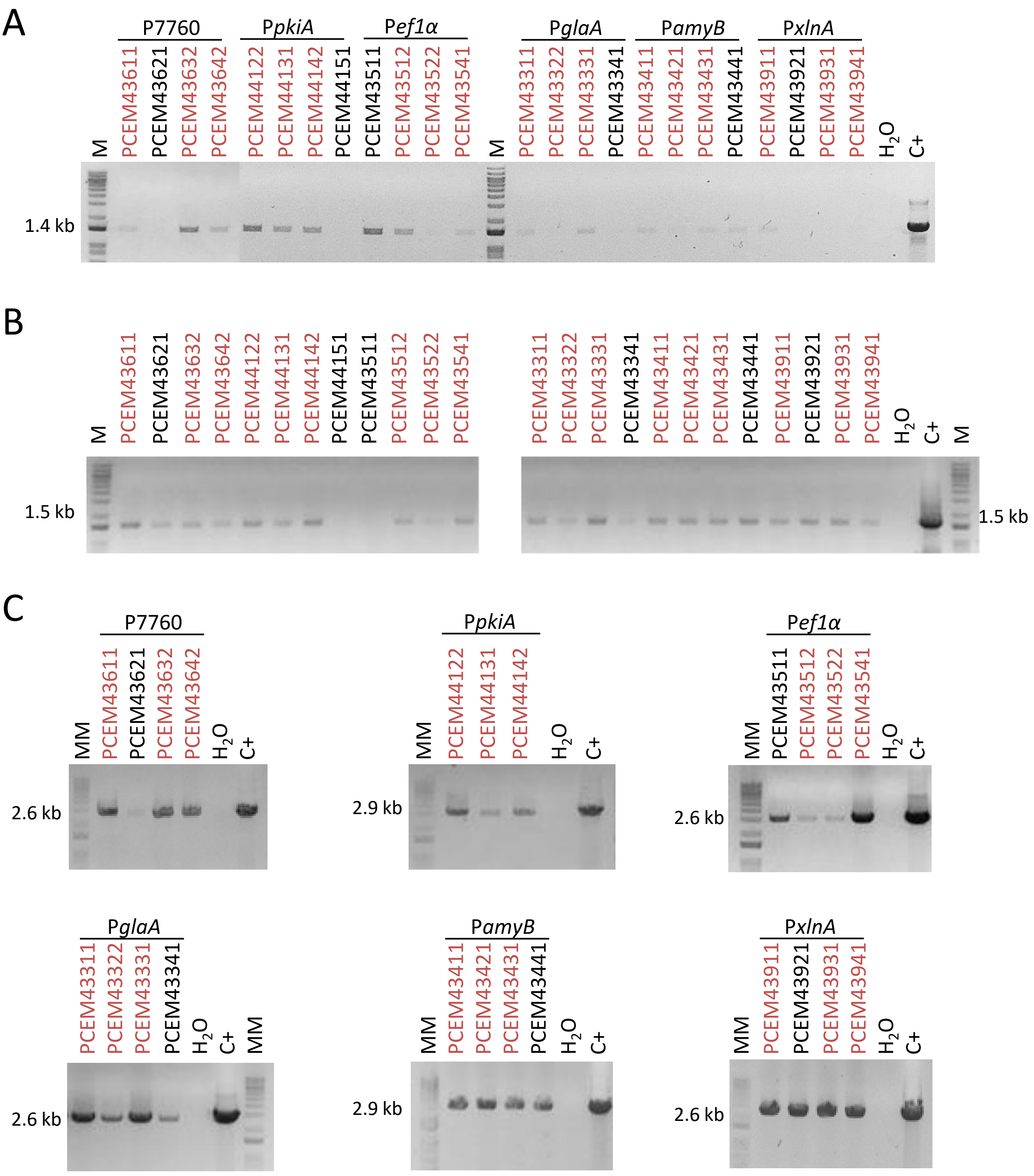

Supp. Figure S2. Molecular characterization of *P. chrysogenum* strains transformed with luciferase reporter system for each of the assayed promoters. (A) Amplification of the *nptII* geneticin resistance TU (1.4 kb) using primers OJM371 and OJM555. (B) Amplification of Nanoluciferase TU (1.5 kb) using primers OJM509 and OJM555. (C) Amplification of luciferase TUs for each construct using the reverse primer OJM522 and the corresponding forward primer for each promoter (OJM705 for P*xlnA*, OJM706 for P*pkiA*, OJM708 for P*glaA*, OJM709 for P*amyB*, OJM710 for P*ef1*α, and OJM711 for P7760). The 2.6 kb bands correspond to the complete luciferase TU expressed under the promoter P*xlnA* (FB439), P*glaA* (FB433), P7760 (FB436) or P*ef1*α (FB435), and the 2.9 kb bands correspond to the luciferase TU under P*amyB* or P*pkiA*. Names in red correspond to the validated transformants that were selected for the luciferase assay.

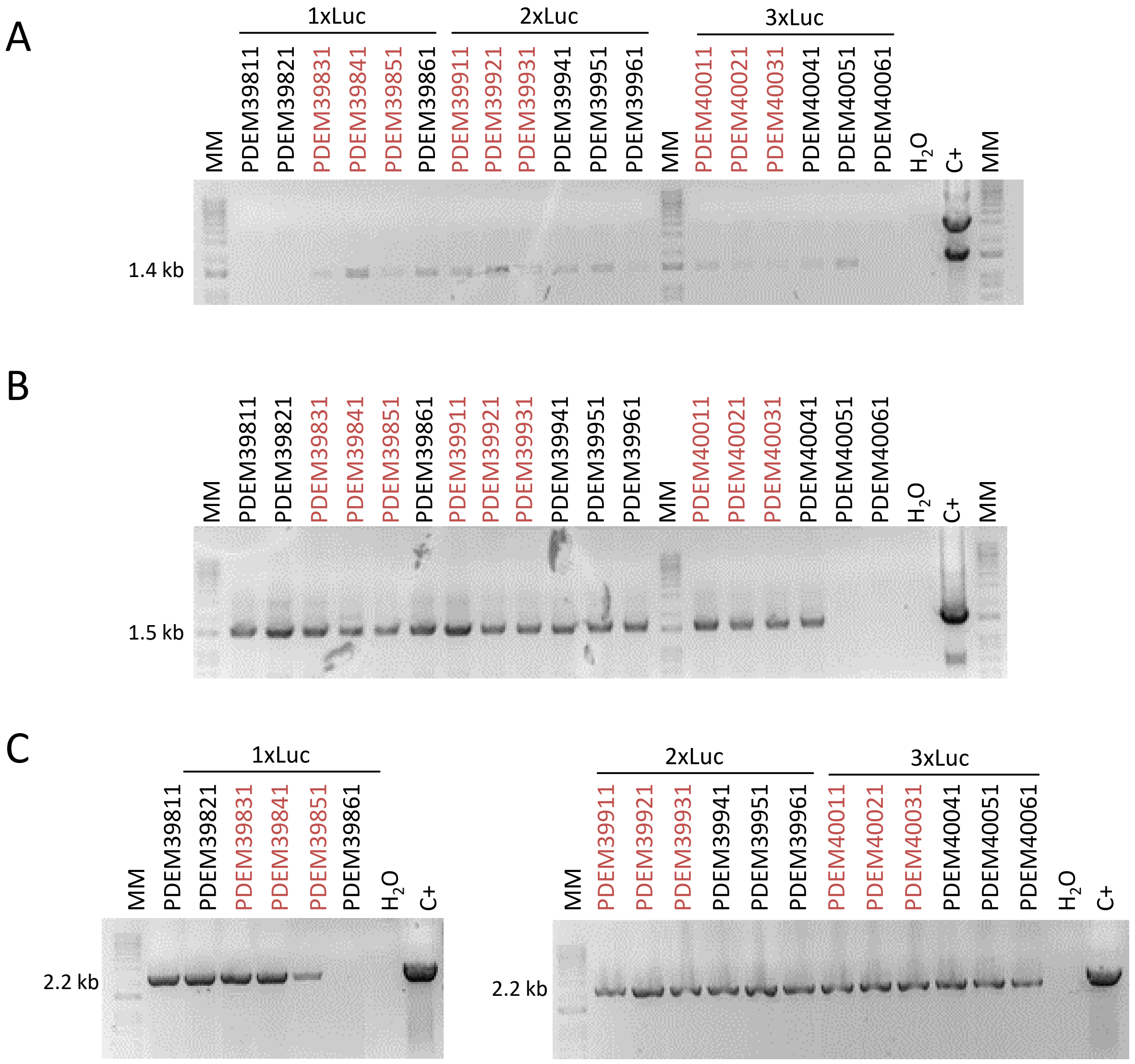

Supp. Figure S3. Molecular characterization of *P. digitatum* strains transformed with luciferase reporter system under the regulation of GB_SynP synthetic promoters. (A) Amplification of the geneticin resistance *nptII* TU (1.4 kb) using primers OJM371 and OJM555. Transformants names correspond to with the construct carried, which contained the luciferase under the regulation of the synthetic promoter with one repetition of the gRNA1 target sequence (1xLuc, FB398 construct, PDEM398XX transformants), two repetitions (2xLuc, FB399, PDEM399XX) or three repetitions (3xLuc, FB400, PDEM400XX). (B) Amplification of Nanoluciferase TU (1.5 kb) using primers OJM509 and OJM555. (C) Amplification of luciferase TU (2.2 kb) in each construct using the primers OJM522 and OJM754 for 1xLuc and the primers OJM522 and OJM755 for 2xLuc and 3xLuc. Names in red correspond to the validated transformants that were selected for the luciferase assay.

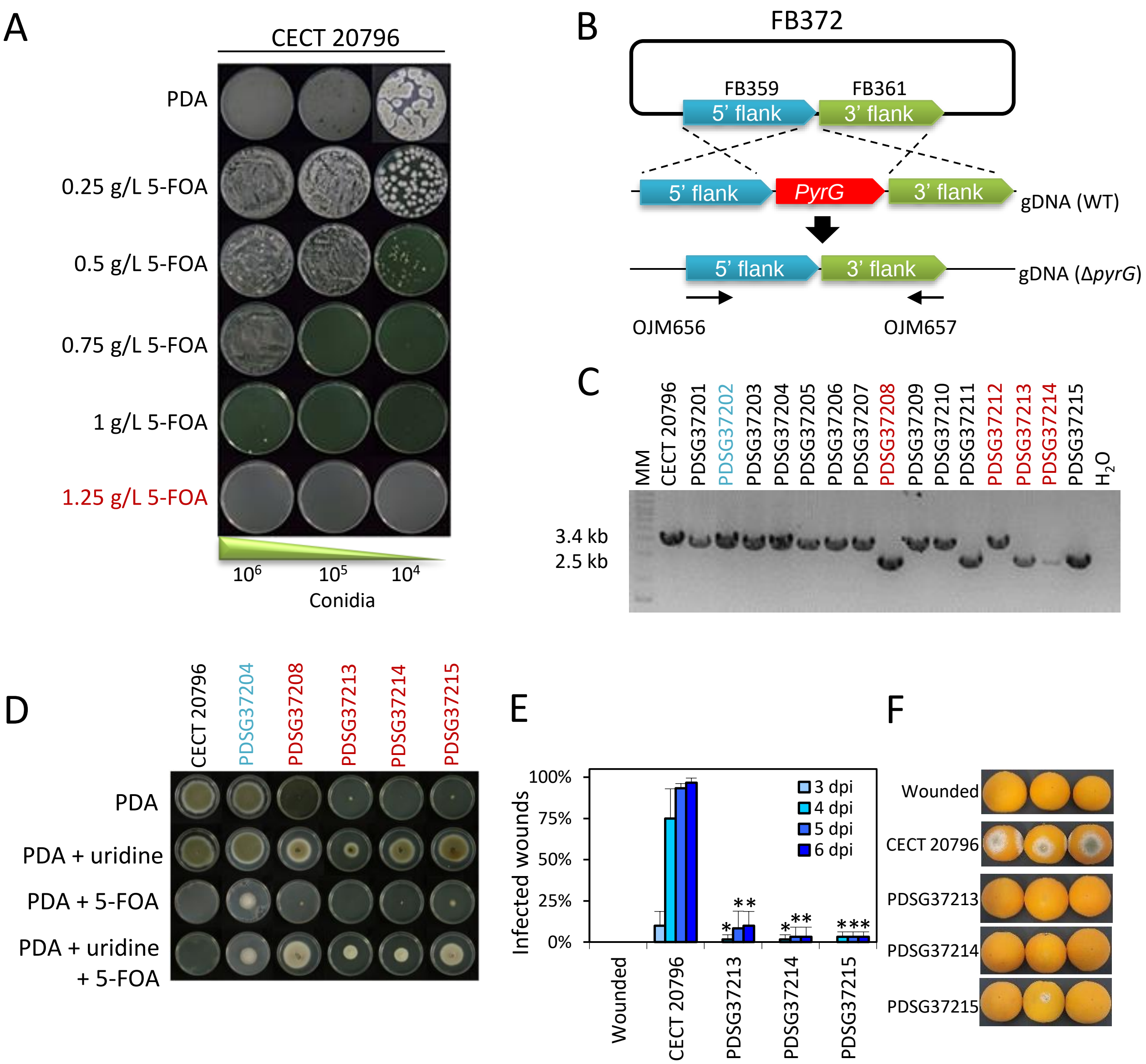

Supp. Figure S4. Generation of *P. digitatum* Δ*pyrG* mutants. (A) Minimum Inhibitory Concentration (MIC) analysis of *P. digitatum* CECT 20796 in the presence of increasing concentrations of 5-Fluoroorotic acid (5-FOA). A concentration of 1.25 g/L was chosen for *pyrG* deletant selection (in red). (B) Plasmid pDGB3α1 FB372 meant for the deletion of *pyrG* gene in *P. digitatum* by ATMT and homologous recombination. Note that no marker was included for positive selection. Primers OJM656 and OJM657 were used for the molecular characterization of the 5-FOA resistant transformants shown in (C). The 3.4 kb band corresponds to the *pyrG* gene whereas 2.5 bands correspond to expected amplicon size after *pyrG* deletion. Selected transformants are highlighted in red. (D) Growth profile of positive Δ*pyrG* transformants. PDSG37024 was included as a control of a spontaneous 5-FOA resistant strain that did not have *pyrG* deleted as shown in (C) (in blue). (E) Fruit infection assays of Δ*pyrG* mutants on oranges. Data indicate the percentage of infected wounds (mean ± SD) at each day post-inoculation (dpi). (*) shows statistical significance between each sample compared to the control CECT 20796 at each dpi (*t* test, $p < 0.05$). (F) Representative images of oranges infected by the indicated strains at 6 dpi.
